# Apologetic

**Published:** 1867-06

**Authors:** 


### *Apologetic.*

The confusion incident to a change of office, and all the annoyances appendant thereto, together with unexpected pressure of other duties, must excuse poverty of the editorial columns the present month. Hereafter, there shall be no cause of complaint on this account.

